# Interventions to strengthen the leadership capabilities of health professionals in Sub-Saharan Africa: a scoping review

**DOI:** 10.1093/heapol/czaa078

**Published:** 2020-12-13

**Authors:** Oliver Johnson, Kerrin Begg, Ann H Kelly, Nick Sevdalis

**Affiliations:** 1 Centre for Implementation Science, Health Services and Population Research Department, Institute of Psychiatry, Psychology & Neuroscience, King’s College London, David Goldberg Centre, De Crespigny Park, London SE5 8AF, UK; 2 Centre for Health Policy, School of Public Health, School of Public Health Building. University of the Witwatersrand, 27 St Andrews Road, Parktown, 2193 South Africa; 3 School of Public Health and Family Medicine, University of Cape Town, Falmouth Building, Anzio Road, Cape Town, 7925, South Africa; 4 Department of Global Health & Social Medicine, School of Global Affairs, Faculty of Social Science & Public Policy, King’s College London, Bush House, North East Wing, 40 Aldwych, London, WC2B 4BG, UK

**Keywords:** Accountability, capacity building, decision making, health workers, management, students, review, physicians, nurses

## Abstract

Leadership is a critical component of a health system and may be particularly important in Sub-Saharan Africa, where clinicians take on significant management responsibilities. However, there has been little investment in strengthening leadership in this context, and evidence is limited on what leadership capabilities are most important or how effective different leadership development models are. This scoping review design used Arksey and O’Malley’s approach of identifying the question and relevant studies, selection, charting of data, summarizing of results and consultation. A comprehensive search strategy was used that included published and unpublished primary studies and reviews. Seven databases were searched, and papers written in English and French between 1979 and 2019 were included. Potential sources were screened against inclusion and exclusion criteria. Data were grouped into common categories and summarized in tables; categories included conceptual approach to leadership; design of intervention; evaluation method; evidence of effectiveness; and implementation lessons. The findings were then analysed in the context of the review question and objectives. Twenty-eight studies were included in the review out of a total of 495 that were initially identified. The studies covered 23 of the 46 countries in Sub-Saharan Africa. The leadership development programmes (LDPs) described were diverse in their design. No consistency was found in the conceptual approaches they adopted. The evaluation methods were also heterogeneous and often of poor quality. The review showed how rapidly leadership has emerged as a topic of interest in health care in Sub-Saharan Africa. Further research on this subject is needed, in particular in strengthening the conceptual and competency frameworks for leadership in this context, which would also inform better evaluation. Our findings support the need for LDPs to be accredited, better integrated into existing systems and to put greater emphasis on institutionalization and financial sustainability from their early development.

**KEY MESSAGES**

- Of the 28 studies identified in this review, 77% were published in the last 5 years, suggesting an emerging interest in the role of leadership development in strengthening health systems in Sub-Saharan Africa.
- There is significant diversity in the conceptual approaches to leadership that underpin leadership development programmes (LDPs) in Sub-Saharan Africa, and many do not appear to be based on a clear theoretical framework at all, highlighting the need for more research on how leadership functions in these contexts and how it can be strengthened.
- The methodological approaches to evaluating LDPs are often weak and there is a need for longer term evaluations that more coherently assess the outputs and outcomes of the programmes as they relate to leadership.
- More emphasis is needed on the long-term sustainability of LDPs in Sub-Saharan Africa, which should ideally be financially viable with domestic resources and delivered by national or regional institutions and faculty.

## Introduction

Leadership is widely recognized to be a critical component of an effective health system (World Health Organization, 2007a). The impact of good leadership across the health system are wide-ranging and catalytic, from improving the performance and retention of the health workforce to ensure the fair distribution and effective expenditure of the health budget and the maintenance of health infrastructure (Swanson *et al.*, 2010). According to Frenk and colleagues, ‘Probably the most complex challenge in health systems is to nurture persons who can (_**…**_) lead the complex processes of policy formulation and implementation’. Put simply, ‘Without leaders, even the best designed systems will fail’ (Frenk, 2010, p. 2).

In the absence of highly formalized health governance systems, where clinical and operational decision-making may be less comprehensively regulated, the need for leadership for health system functioning and routine clinical practice is perhaps even more keenly felt. Many Sub-Saharan African countries, for example, lack a health management cadre and thus depend upon individual health professionals to take on day-to-day management responsibilities at the facility, district or national level (World Health Organization, 2010). These organizational demands are compounded by the critical shortage of health workers, resulting in particularly capable individuals taking on significant responsibilities at an early stage in their career. The multiple and critical leadership roles individuals play can mean that he or she has more scope to affect outcomes through their decisions and behaviour, thus amplifying the importance of leadership capabilities within health professional groups (Kruk *et al.*, 2018, pp. e1232–3).

Enhancing leadership has received increasing emphasis by health professionals in high-income countries, where specific medical leadership frameworks have been developed (NHS, 2010; Frank *et al.*, 2015). However, despite evidence of the importance of effective leadership and management on health system performance, (World Health Organization, 2007b; Curry *et al.*, 2012; Swensen *et al.*, 2013), leadership has received comparatively little investment in low- and middle-income countries, precisely the contexts where the development of leadership capabilities are needed most (World Health Organization, 2008). Contributing to this challenge is a lack of clear evidence of exactly what leadership capabilities are most important for health professionals in Sub-Saharan Africa to be effective in their roles, or how effective different models and approaches to leadership development programmes (LDPs) have been.

The aim of this study was to address the above gap through a scoping review of the literature on LDPs for health professionals in Sub-Saharan Africa. The review was systematically carried out to address three specific objectives. Firstly, to describe existing LDPs in terms of their conceptual approach to leadership and programme design. Secondly, to describe the range of available literature on this subject and identify gaps. Finally, to analyse the lessons learnt from previous LDPs in this context.

## Methods

**Figure 1:**
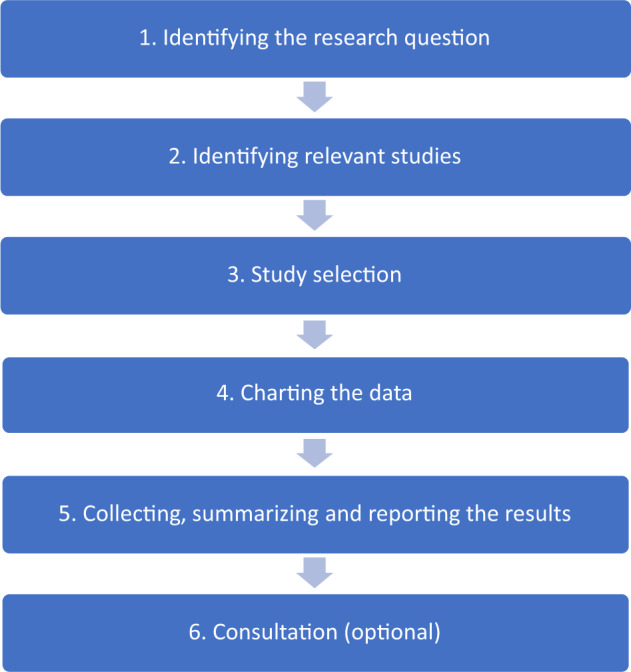
Six stages of a scoping review (Arksey and O’Malley, 2005).

The study followed standard scoping review methodology *(*Figure 1; Arksey and O’Malley, 2005)—described in the following sections.

**Figure 2:**
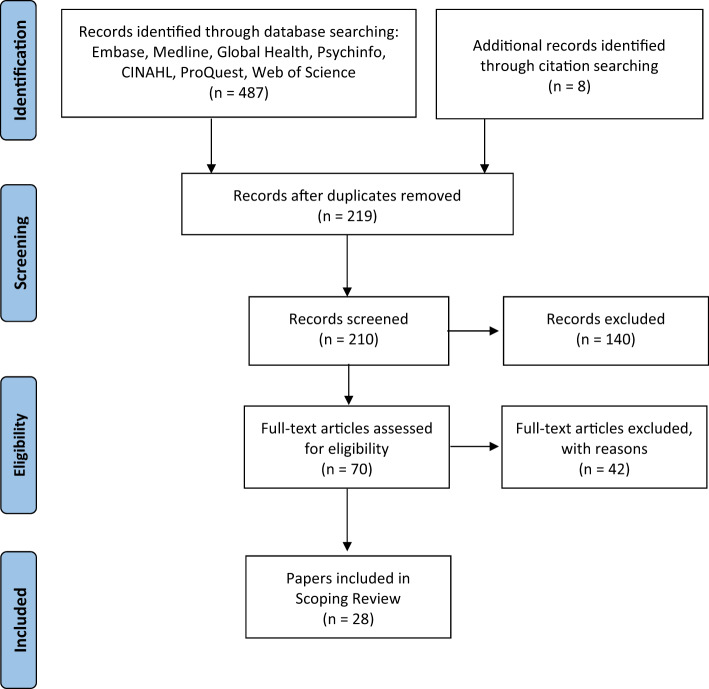
Flow diagram of selection of studies included in the review.

### Identifying the research question

The population, concept, context framework are a common tool used to construct research questions for scoping reviews (Peters *et al.*, 2015). Here, the ‘population’ was defined as health professionals and the ‘concept’ as interventions to strengthen leadership capabilities. No single definition, framework or conceptualization of leadership or leadership capabilities was selected for this review, so as to leave the scope broad and to explore how these were framed in the studies. The context was understood as relating to any of the World Health Organization health system building blocks, including leadership, health workforce and training or service delivery, in Sub-Saharan Africa (World Health Organization, 2007a).

### Identifying relevant studies

A comprehensive search strategy was used that included published primary studies, unpublished (grey literature) primary studies and reviews. The search string is listed in Box 1. The following databases were used for the search: MedlinePlus, Embase, PsycINFO, Global Health, CINAHL, ProQuest, and Web of Science. Papers written in English and French were included, which meant that the majority of relevant literature should have been captured, as almost all academic papers relating to Sub-Saharan Africa are published in these two languages. French papers had to have an abstract written in English. The search covered papers published between 1 January 197*9 (*The date of publication of the seminal book ‘Leadership’ by James McGregor Burns that was a key foundation to modern leadership theory (MacGregor Burns, 1978), and 31 November 2018.

**Box 1:** Eligibility criteria and search string

Inclusion criteria	Exclusion criteria
Published on/after 1978 Describes a previous or existing leadership development intervention Targets health professionals as participants Includes at least one country in Sub-Saharan Africa Published peer-reviewed journal, technical report or academic thesis Written in English; or French with English abstract available	Published before 1978 Does not include a leadership intervention, or intervention not yet delivered Does not specifically target or reference health professionals Does not include at least one country in Sub-Saharan Africa Conference abstract without full text available No English abstract available
**Search string** (Africa* OR Angola* OR Benin* OR Botswana OR Batswana OR ‘Burkina Faso’ OR Burkinese OR Burundi* OR Cameroon* OR ‘Cape Verd*’ OR ‘Cabo Verd*’ OR ‘Central African Republic’ OR Chad* OR Comoros OR Comoran OR Congo* OR ‘Democratic Republic of Congo’ OR ‘Equatorial Guinea*’ OR Eritrea* OR Ethiopia* OR Gabon* OR Gambia* OR Ghana* OR Guinea* OR ‘Guinea Bissau’ OR ‘Ivory Coast’ OR ‘Cote d’Ivoire’ OR Kenya* OR Lexsotho OR Basoth* OR Liberia* OR Madagasca* OR Malawi* OR Mali* OR Mauritania* OR Mauretania* OR Maurit* OR Mayot* OR Mozambiq* OR Mocambiq* OR Namibia* OR Niger* OR Rwanda* OR ‘Sao Tome’ OR Senegal* OR Seychell* OR ‘Sierra Leone*’ OR Somali* OR ‘South Africa*’ OR Sudan* OR Swazi* OR eSwatini OR Tanzania* OR Togo* OR Uganda* OR ‘Western Sahara*’ OR Zaire OR Zambia* OR Zimbabwe*) AND (Health* OR medic* OR clinic* OR hospital* OR ‘primary care’ OR doctor* OR physician* OR nursing OR nurse* OR midwife* OR pharmacist* OR pharmacy OR dental OR dentist* OR psychiatrist* OR psychologist* OR surgeon*) AND (leader*) NOT (‘local leader*’ or ‘opinion leader*’ OR ‘peer health leader*’ OR ‘traditional leader*’ OR ‘faith leader*’ or ‘government leader*’ OR ‘community leader*’ OR ‘religious leader*’ OR ‘muslim leader*’ OR ‘christian leader*’ OR ‘African American’)	

### Study selection

The eligibility criteria for inclusion in the review are summarized in Box 1.

A total of 487 papers were initially identified by the search, with an additional eight papers picked up through citation search. After duplicates were removed and records screened, 70 papers were assessed for eligibility of which 28 papers were included in the final review (see Figure 2). These represent 27 different LDPs *(*two papers reported on the same LDP: Kebede *et al.*, 2010, 2012).

### Charting the data

Charting (i.e. extracting) the data was done using a structured Microsoft Excel spreadsheet with 31 fields that were clustered under the following headings: details of publication; research question; conceptual approach to leadership; design of intervention; evaluation method; evidence of effectiveness; implementation lessons; and reviewers’ decision.

The charting spreadsheet was developed and piloted with one study by the lead author, and subsequently reviewed and refined across all authors. Charting was then completed by the lead author, referring any ambiguous or uncertain issues to the review team until consensus was achieved.

### Collating, summarizing and reporting the results

The charted data were split into key thematic sections, including programme structure, learning content, learning methods and evaluation. Data were grouped into common categories and summarized in tables. The findings were then analysed in the context of the overall review question and specific objectives.

### Consultation

A consultation on the review findings will form part of subsequent research.

### Preferred reporting items for systematic reviews and meta-analyses extension for scoping reviews

The review met the 27 criteria set out by the Preferred reporting items for systematic reviews and meta-analyses Extension for Scoping Reviews (Tricco *et al.*, 2018), such as inclusion of an explicit statement of the questions and objectives, the eligibility criteria, a flow diagram of evidence screened discussions of the limitations of the scoping review process. The study protocol was registered online with the Open Science Framework. (https://osf.io/c7rwf/)

## Results

Twenty-eight studies were identified and included in our review, of which 20 (77%) were published in the last 5 years (2014–18) and none was published before 2002, despite the literature search covering a 40-year span (1979–2018).

LDPs were undertaken in 23 of the 46 countries in Sub-Saharan Africa, representing half of the region. Some countries were represented across multiple studies, and the number of different LDPs carried out in each country is shown in Box 2. Five countries (South Africa, Uganda, Ethiopia, Kenya and Zambia) account for 52% of the countries where LDPs have been developed, implemented and evaluated in the published evidence base. We found one or two studies carried out in each of the remaining 18 countries (Box 2).

**Box 2:** Frequency of Sub-Saharan African countries that were included in studies

South Africa	8	Cameroon	1
Uganda	8	Eswatini	1
Ethiopia	4	Gambia	1
Kenya	4	Lesotho	1
Zambia	3	Liberia	1
Botswana	2	Mauritius	1
Ghana	2	Namibia	1
Malawi	2	Nigeria	1
Mozambique	2	Seychelles	1
Rwanda	2	Sierra Leone	1
Tanzania	2	South Sudan	1
Zimbabwe	2		

The results are organized into three sections, starting with a breakdown of the LDPs themselves. This includes their target group, length, conceptual approach to leadership, intended outcomes, learning content and the teaching and learning methods. The next section looks at how the LDPs were evaluated for effectiveness, as well as the key findings from those evaluations. Finally, the lessons learnt from the experience of running the LDPs or from their formal evaluation are summarized.

### Leadership development programmes

The 27 LDPs are summarized in Table 1, ordered by year of publication. The countries where the LDPs took place and key components of the overall structure of the LDP are listed. The table also reports descriptive information on the type and nature of the LDP reviewed, including who the LDP targets, its capacity in participant numbers, its length of delivery and whether it leads to a formal qualification upon successful completion.

**Table 1:** Summary of the studies and the structure of the leadership development programmes, listed in order of publication year

Author	Country	Context	Target group of participants	Number of participants	Length	Qualification
Dovey (2002)	South Africa	On-the-job programme	Interdisciplinary (district health management teams)	12–20 in each team	2 years	Certificate in district health management
Perry (2008)	Mozambique	On-the-job programme	Interdisciplinary (district health management teams)	Approx. 25	2 years	
Kebede *et al.* (2010, 2012)	Ethiopia	On-the-job programme	Medical doctors (newly appointed CEOs)	24 per cohort	2 years	Masters of hospital and healthcare administration
Matovu *et al.* (2011)	Uganda	Training programme followed by placement	Interdisciplinary (HIV practitioners from clinical and management backgrounds)	77 fellows over 8 years	2 years	
Seims *et al.* (2012)	Kenya	On-the-job programme	Interdisciplinary (district or facility managers including medical doctors, nurses and administrators)	67 teams	6 months	
Abdulmalik *et al.* (2014)	The Gambia, Ghana, Liberia, Nigeria, Sierra Leone	Short course	Interdisciplinary (mental health practitioners including clinicians and reps from government and civil society)	106 over 4 cohorts (21 per cohort)	2 weeks	
Kwamie *et al.* (2014)	Ghana	On-the-job programme	Interdisciplinary (district and facility managers)	4–7 per team	6 months	
Nakanjako *et al.* (2015)	Uganda	Training programme followed by placement	Interdisciplinary (nurses and medical doctors)	14 over 4 years (4 per cohort)	1 year	
Wilson *et al.* (2015)	South Africa	On-the-job programme	Interdisciplinary (district, sub-district and facility managers)	400 over 6 years	6 months	
Downing *et al.* (2016)	Uganda	On-the-job programme	Nurses (palliative care nurses)	20 per cohort	2 years	
Edwards *et al.* (2015)	Jamaica, Kenya, Uganda, South Africa	On-the-job programme	Interdisciplinary (HIV nurses, researchers and reps of government and civil society)	167 in 12 hubs	5 years	
Goldstone and Ntuli, (2016)	South Africa	Fellowship with part-time postgraduate degree course	Interdisciplinary (provincial, district and facility managers)	178 fellows over 4 years (94 enrolled PGDip, 75 enrolled MPH)	1–2 years	Postgraduate diploma or Masters in public health
Najjuma *et al.* (2016)	Uganda	Training programme followed by placement	Interdisciplinary (undergraduate health sciences students)	242 per cohort	6 weeks	Undergraduate module
Ousman *et al.* (2016)	Botswana, Kenya, Uganda, Tanzania, USA	Training programme followed by placement	Interdisciplinary (HIV professionals including medical doctors, nurses, public health specialists and pharmacists)	100 over 6 years	1 year	
Footer *et al.* (2017)	Ethiopia	On-the-job programme	Physiotherapists	17 per cohort	4 years	Doctor of physiotherapy
Kvach et al. (2017)	Ethiopia	International fellowship	Interdisciplinarty (female health sciences faculty)	9 per cohort	2 weeks	
Mutale *et al.* (2017)	Zambia	On-the-job programme	Interdisciplinary (district health managers)	767 over 2 years	6–2 months	Diploma in management and leadership
Szabo *et al.* (2017)	South Africa	On-the-job programme.	Interdisciplinary (mental health practitioners)	15	2 years	
Aagaard *et al.* (2018)	Zimbabwe	On-the-job programme	Interdisciplinary (health sciences faculty)	42 over 3 cohorts (14 per cohort)	1 year	
Bates *et al.* (2018)	Malawi	Short course	Interdisciplinary (from diverse, health-related settings)	21 per cohort	5 days	
Cleary *et al.* (2018)	South Africa	On-the-job programme	Interdisciplinary (district and facility managers, mostly from nursing backgrounds)	15 per cohort	4 years	
Doherty *et al.* (2018)	South Africa	On-the-job programme	Interdisciplinary (senior public health managers)	91 over 6 cohorts (15 per cohort)	18 months	Postgraduate diploma in health management
Dzudie *et al.* (2018)	Cameroon	Conference	Interdisciplinary (HIV clinicians and researcher)		1 day	
Foster *et al.* 2018)	Zambia	On-the-job programme	Nurses (district and facility managers)	23 per cohort	1 year	Certificate in leadership and management practice
Gross *et al*. (2018)	17 Countries in SSA	On-the-job programme	Nurses and midwives (from ministry, academia and associations)	4 per country	3–4 years	
Muhimpundu *et al.* (2019)	Rwanda	Short course	Interdisciplinary (NCD programme managers)	14 in the cohort	4 days	
Spies *et al.* (2018)	Uganda	Short course	Nurses	18	2 days	

#### Target group and size of the LDPs

Twenty-one of the 27 LDPs (78%) were targeted at interdisciplinary groups. This included district and facility management teams (33% of all LDPs), HIV professionals (15%), mental health professionals (7%), health sciences faculty (7%), undergraduate health sciences students (4%), public health managers (4%) and non-communicable disease programme managers (4%). Of the remaining six LDPs, 4 (15%) were targeted at nurses/midwives (including specifically palliative care nurses in one case), 1 (4%) was targeting medical doctors who were newly appointed hospital chief executives and 1 (4%) was aimed at physiotherapists.

The majority (68%) had 10–25 participants in each cohort, with 4 (15%) having 4–9 participants. Larger courses were rare.

#### Length, context and accreditation of the LDPs

Seven LDPs (26%) were shorter than 6 months. Of these, three LDPs were shorter than a week, including the hypertension training by Spies *et al.* (2018) that was 2 days (L Spies, personal communication, 15 July 2019). Most (60%) of the LDPs were between 6 months and 2 years in length. The remaining four LDPs (15%) were longer than 2 years.

Only 8 (30%) of the LDPs we reviewed provided a formal qualification to delegates completing them successfully. Najjuma *et al.* (2016) describe an undergraduate health sciences module in Uganda. The LDPs referred to by Dovey (2002)in South Africa and Foster *et al.* (2018) in Zambia both provided Certificates (in Health Management and in Leadership and Management Practice, respectively). Mutale *et al.*’s (2017) LDP in Zambia led to a Diploma in Management and Leadership, while Doherty *et al.*’s (2018) in South Africa led to a Postgraduate Diploma in Health Management. In Goldstone and Ntuli, (2016) ASELPH programme, participants could graduate with a Postgraduate Diploma or a Masters in Public Health. Kebede *et al.*’s (2010, 2012) LDP for newly appointed CEOs in Ethiopia was a Masters of Hospital and Health Care Administration. Finally, Footer *et al.*’s (2017) LDP was a Doctorate in Physiotherapy in Ethiopia.

The majority (60%) of the LDPs took the form of an on-the-job programme, where participants remained in their current roles and received additional leadership development support. Seven LDPs (26%) took people away from their current roles for short periods: one was a conference session; one was a brief international fellowship; one included modular course work; and four were short courses. The remaining four studies (15%) consisted of a training programme followed by a placement in a host organization. Of those, one was an undergraduate module and three were fellowships for health professionals.

Detailed review is offered in the sections that follow, accompanied by in-depth analysis of the leadership conceptualizations and frameworks that underpin the LDPs.

A summary of three of the LDPs is provided in Box 3, selected to show the diversity between the programmes in terms of participants, structure and conceptual approach. The summaries give an overview of how the LDPs could look in practice.

#### Conceptual approaches to leadership

Only seven studies (26%) explicitly stated the LDP’s conceptual approach to leadership. Definitions for these conceptual approaches are summarized in Box 4. Spies *et al.*’s (2018) LDP on nurse education in Uganda and Najjuma *et al.*’s (2016) undergraduate training programme in Uganda both adopted the individualized transformational concept of leadership. Dovey’s (2002) LDP in South Africa also adopted a transformational approach to leadership but combined this with a distributed concept of leadership, where leadership is shared across a team or group, showing that these concepts are not mutually exclusive but can be coupled together in different ways. Dzudie *et al.*’s (2018) research leadership training in Cameroon adopted a collective approach to leadership. Finally, Cleary *et al.*’s (2018) programme on leadership in primary health care, Doherty’s (2018) study on the OR Tambo Fellowship, and Wilson’s (2015) Wellness for Effective Leadership programme in South Africa all described a distributed approach to leadership that is specifically relational.

Box 3 Case studies of three LDPs from the scoping review

**The Ethiopia Hospital Management Initiative (EHMI) (Kebede *et al.*, 2010, 2012).**

The EHMI was a large-scale initiative established by the Federal Ministry of Health in Ethiopia to strengthen hospital management capacity across the country. It took a comprehensive approach that included the restructuring of senior management roles in government hospitals by creating the post of Chief Executive Officer (CEO), the development of ‘Standards for hospital management in Ethiopia’ and ‘the Blueprint for hospital management in Ethiopia’ and the introduction of a 2-year executive-education style Masters of Healthcare and Hospital Administration (MHA) programme.

The MHA was targeted at new hospital CEOs and was hosted by Jimma University in Ethiopia with initial support from Yale University in the USA. The course consisted of a series of 3-week blocks, every 4 months, with participants working in their hospitals the rest of their time. There were 25–30 participants in each cohort. The learning content included public health, health policy, problem-solving, supply chain management, hospital operations, healthcare financial management, strategic management, nursing management, human resource management and leadership development. When back at their hospitals, participants sent weekly progress reports to their faculty and received on-site supervision.

**Multidisciplinary leadership training for undergraduates at Mbarara University of Science and Technology (MUST) in Uganda (Najjuma *et al.*, 2016)**

When the Ugandan Ministry of Health identified the need to strengthen the leadership and management skills of health professionals in the country, MUST decided to introduce a leadership training programme for all undergraduates studying a Bachelor’s degree in nursing, medicine and surgery, pharmacy, and medical laboratory science.

The 250 undergraduate health sciences students were given 1 week of teaching in leadership theory before being allocated to multidisciplinary teams of 7–10 people. Each team was then placed in a rural community for 5 weeks, with the objective of working with that community to identify a health-related problem where they could intervene.

**Wellness for Effective Leadership (WEL) in South Africa (Wilson *et al.*, 2015)**

The WEL programme was undertaken by over 400 frontline managers in the public sector in South Africa from 2009 to 2014, aimed at building emotional intelligence and developing personal and interpersonal competencies. It was based on the premise that ‘at the core of transformation of any service are the individuals who run the services, and that the changes brought about through greater self-awareness and self-care, perceptions of well-being and reduced stress, lead to an increased ability to manage stressful situations and conflicts’.

The standard programme consisted of three 2-day workshops, followed by a final 1-day workshop, with a 6–8-week gap between each to allow for reflection and experimentation. The workshops began with participants reflecting on their own life’s journey and contexts, and included a screening for compassion satisfaction, risk of burnout and secondary traumatic stress. The second and third workshop were then tailored to address the major issues that had been identified, before a final workshop where participants would report back to colleagues, family members and visitors.

**Box 4:** Definitions of conceptual approaches to leadership Referenced in the Studies

Individualized	Effective performance by an individual, group, or organization is assumed to depend on leadership by an individual with the skills to find the right path and motivate others to take it (Yukl, 1999, p. 292)
Pluralized	Leadership as an emergent network of relations, which is a shared phenomenon, encompassing several leaders who may be both formally appointed and emerge more informally (White *et al*., 2016, p. 280)
Distributed/collective	Sub-categories of pluralized leadership where leadership functions may be shared by several members of a group, allocated to individual members or performed by different people at different times (Yukl, 1999, p. 292)
Transformational	The process whereby a person engages with others and creates a connection that raises the level of motivation and morality in both the leader and the follower (Northouse, 2015 p. 164)
Relational	Leadership as an interpersonal phenomenon associated with collaboration, empathy, trust and empowerment [Cummings *et al*., 2010in Cleary *et al.* (2018)]

Eleven other LDPs (41%) appeared to adopt a particular approach to but this was not made explicit in the studies. Five LDPs (19%) had an implied focus on individual leadership (Kebede *et al.*, 2010; Nakanjako *et al.*, 2015; Goldstone and Ntuli, 2016; Ousman *et al.*, 2016; Footer *et al.*, 2017). The studies of three LDPs (11%) inferred an individualized approach to leadership but also emphasize the importance of working in teams (Mutale *et al.*, 2017; Szabo *et al.*, 2017; Bates *et al.*, 2018). Three studies of the LDPs (11%) referred to team-based leadership, suggesting a pluralized concept (Perry, 2008; Seims *et al.*, 2012; Kwamie *et al.*, 2014).

In summary, of the 18 LDPs (67%) where a concept of leadership was explicit or could be implied, 5 (19%) emphasized individualized leadership, 5 (19%) mixed individualized and pluralized concepts and 6 (22%) focused on pluralized approaches. This range and spread suggest that there is no consensus on how leadership functions or is generated in the context of health care in Sub-Saharan Africa. We explore the implications of this finding in the Discussion.

#### Use of leadership frameworks

Eight studies (30%) made specific reference to a leadership framework on which the reported LDP is based. Three LDPs (11%) used competency frameworks. Foster *et al.*’s (2018) paper on nurse leadership in Zambia drew on a list of 14 competencies for facility heads in primary care in Zambia. Muhimpundu *et al.*’s (2019) leadership and management training for non-communicable disease programme managers in Rwanda was guided by competencies drawn from public health competency frameworks in the USA. Nakanjako *et al.*’s (2015) study from Uganda described an LDP that used a competency-based curriculum to provide participants with ‘practical leadership and management skills’.

One LDP (4%), Dzudie *et al.*’s (2018) research leadership training in Cameroon, referred to leadership competence but did not elaborate further. Similarly,  Goldstone and Ntuli, (2016) study referred to a set of 14 leadership competencies that were not specified in the paper. Two LDPs (7%), Seims *et al.*’s (2012) in Kenya and Perry (2008) in Mozambique, were based on the Management Sciences for Health Leadership and Management Framework. This framework consists of a list of practices under four domains of leading (scanning, focusing, aligning/mobilizing and inspiring) and four domains of managing (planning, organizing, implementing and monitoring and evaluating). One LDP (4%), Mutale *et al.*’s (2017) from Zambia, referred repeatedly to ‘leadership and management knowledge and skills’, but designed its evaluation around competency frameworks from the UK’s NHS Leadership Academy. One LDP (4%), Cleary *et al.*’s (2018) about primary health care teams in South Africa, was based on a mix of behaviours, attitudes, values and beliefs drawn from the ‘Thinking Environment’ model. Similarly, Doherty’s (2018) study on the OR Tambo fellowship similarly referred to ‘knowledge, skills, attitudes and behaviours’. Spies *et al.*’s (2018) hypertension workshop drew on Drenkard’s (2012) Conceptual Framework for Leadership.

The remaining studies did not specifically state whether the approach of the LDP was based on a leadership framework, although the approach could be inferred from the content of the LDP and how it was evaluated (see later section).

#### Intended outcomes and theories of change of the LDPs

Eighteen studies (67%) set out the outcomes or goals they expect from the LDP. Trying to untangle this information and fit it into strict categories of outputs, outcomes and goals was difficult as this can be framed in different ways. Therefore, this review has taken a slightly flexible approach and focused on drawing useful conclusions.

An expressed outcome or goal of most of the LDPs was improved health service delivery or performance, either in general or for a specific programmatic area, and this was referred to in 14 (78%) of those 18 LDPs. Some components of performance were specifically identified, including safety (Foster *et al.*, 2018), sustainability (Matovu *et al.*, 2011) and evidence-informed practice (Edwards *et al.*, 2015; Muhimpundu *et al.*, 2019). Two studies (7%) referred to mobilizing or making better use of resources (Kwamie *et al.*, 2014; Nakanjako *et al.*, 2015). Najjuma *et al.* (2016) went beyond health service delivery and referred to creating a better and healthier community. Bates *et al.* (2018) referred to creating a more resilient and responsive health system.

Three LDPs (11%) made specific reference to advocacy and policy reform, two of which were focused on mental health and one on HIV (Abdulmalik *et al.*, 2014; Ousman *et al.*, 2016; Szabo *et al.*, 2017). Abdulmalik *et al.* (2014) also identified reduced stigma around mental health as a specific goal of their LDP. Mutale *et al.* (2017) had a specific outcome of improving the workplace climate, while Nakanjako *et al.* (2015) took this a step further and aimed towards increased health worker retention, by reducing migration out of Africa through improved career opportunities.

Finally, three LDPs (11%) included outcomes aimed at the individual participant. Downing *et al.* (2016) aimed to support personal growth, while the hypertension programme of Spies *et al.* (2018) aimed to improve the health habits of participants, as well as to be role models and educators for peers and patients on hypertension. Wilson *et al.* (2015) aimed to enhance self-awareness, self-care and personal well-being, thereby increasing the ability to manage stressful situations and conflicts, impacting positively on productivity, teamwork and service delivery performance.

Six of the studies (22%) described an explicit theory of change for the LDPs—that is to say, they set out the expected outputs, outcomes and goal of the LDP in a clear way (Seims *et al.*, 2012; Abdulmalik *et al.*, 2014; Nakanjako *et al.*, 2015; Wilson *et al.*, 2015; Najjuma *et al.*, 2016; Mutale *et al.*, 2017). Only one study (Edwards *et al.*, 2015) made explicit reference to a ‘theory of change’, although this did not actually meet the generally accepted definition for a theory of change as it set out the expected outputs and outcomes but not a goal.

#### Learning content of the LDPs

The learning content covered by the LDPs, as described in the studies, was grouped into 14 categories, which are summarized in Table 2. Definitions for these categories as they apply to this review are listed in Supplementary Appendix S1.

**Table 2:** Learning content of the leadership development programmes

	Concepts or experiences of leadership	Change management or quality improvement	Project management	Communication	Technical, public health or health systems topics	Policy, influence and advocacy	Relationship management	Self-reflection	Supervision	Strategy development	Ethics or professional values	Network building	Workplace practice	Systems thinking
Dovey (2002)	✓								✓	✓				✓
Perry (2008)	✓	✓		✓			✓		✓					
Kebede *et al.* (2010, 2012)	✓	✓	✓		✓	✓				✓	✓			
Matovu *et al.* (2011)		✓	✓	✓	✓	✓				✓				
Seims *et al.* (2012)	✓													
Abdulmalik *et al.* (2014)				✓	✓									
Kwamie *et al.* (2014)	✓	✓	✓			✓	✓		✓	✓			✓	
Nakanjako *et al.* (2015)	✓	✓	✓	✓	✓									
Wilson *et al.* (2015)				✓			✓	✓	✓			✓	✓	
Downing *et al.* (2016)	✓		✓	✓		✓	✓	✓	✓					
Edwards *et al.* (2015)	✓		✓											
Goldstone and Ntuli, (2016)	✓					✓								
Najjuma *et al.* (2016)														
Ousman *et al.* (2016)	✓	✓	✓	✓										
Footer *et al.* (2017)	✓				✓	✓	✓	✓			✓			
Kvach *et al.* (2017)	✓	✓	✓	✓	✓			✓	✓			✓		
Mutale *et al.* (2017)		✓	✓							✓				
Szabo *et al.* (2017)	✓	✓	✓		✓	✓					✓			
Aagaard *et al.* (2018)		✓	✓		✓		✓	✓	✓					
Bates *et al.* (2018)	✓							✓						
Cleary *et al.* (2018)							✓						✓	
Doherty *et al.* (2018)		✓				✓				✓		✓		✓
Dzudie *et al.* (2018)	✓		✓	✓			✓							
Foster *et al.* (2018)		✓	✓											
Gross *et al.* (2018)							✓							
Muhimpundu *et al.* (2019)		✓			✓	✓								
Spies *et al.* (2018) ^a^	✓			✓	✓			✓						
Total	16	13	13	10	10	9	9	7	7	6	3	3	3	2

a Supplementary information from L Spies, personal communication, 15 July 2019.

Only three categories of learning content appeared in more than half of LDPs, showing the diverse and heterogeneous nature of these programmes to date. The most common was concepts or experiences of leadership, which was referenced in 15 LDPs (56%). In some cases, this consisted of a summary of particular leadership theories or frameworks, while in others, it emphasized reflections on personal leadership experiences of speakers. LDPs also commonly included learning content on project management (48%) and change management or quality improvement (48%).

We included a broad category of technical, public health or health systems topics (33%) to capture a range of quite specific topics that appeared in some LDPs, such as curriculum development (Aagaard *et al.*, 2018), stigma of mental illness (Abdulmalik *et al.*, 2014), biostatistics (Kebede *et al.*, 2010; Matovu *et al.*, 2011) or an update on HIV/AIDS (Nakanjako *et al.*, 2015). These topics were grouped together because they were considered specifically technical, rather than relating to broader leadership or management issues, and often related to the particular context in which the LPD was taking place in. The frequency with which technical topics were included in LDPs suggests the perceived importance of having subject-matter expertise, such as specific clinical knowledge or data analysis skills, when practising leadership in health care, alongside leadership capabilities. The integration of technical expertise may stem from a conceptualization of leadership that emphasizes the importance of technical expertise alongside strategic planning, operational management and relational capabilities. It may also speak to a desire for a hybrid identity in health professional leadership, where perceived ‘leaders’ are considered insiders by both the technical members of their community (such as clinicians, educators or researchers) and those from a management or operational background (Mcgivern *et al.*, 2015).

#### Teaching and learning methods of the LDPs

A similar approach was taken to summarize the teaching and learning methods of the LDPs, as described in the studies. Thirteen categories were identified, with an additional three types of participant assessment. These are summarized in Table 3. The participant assessment refers to any methods used as part of the LDP to assess individual participants as part of their learning and should be distinguished from the programme evaluation described in the next section.

**Table 3:** Teaching and learning methods of the leadership development programmes

	Lectures/workshops	Real world project work	In person mentoring, coaching or supervision	Classroom group work	Peer-mentoring/teaching	Distance mentoring	Placements	Grants	Online module	Newsletter	Workbook	App	Networking	Presentations	Thesis/report	Exam
														Participant assessment		
Dovey (2002)	✓	✓	✓											×	×	
Perry (2008)	✓	✓	✓	✓												
Kebede *et al.* (2010, 2012)	✓	✓		✓										×	×	×
Matovu *et al.* (2011)	✓	✓	✓		✓		✓							×	×	
Seims *et al.* (2012)	✓	✓	✓													
Abdulmalik *et al.* (2014)	✓			✓												
Kwamie *et al.* (2014)	✓	✓	✓													
Nakanjako *et al.* (2015)	✓	✓	✓					✓	✓							
Wilson *et al.* (2015)	✓			✓												
Downing *et al.* (2016)	✓		✓			✓										
Edwards *et al.* (2015)	✓	✓						✓		✓						
Goldstone and Ntuli, (2016)	✓	✓	✓	✓	✓				✓							
Najjuma *et al.* (2016)	✓	✓					✓									
Ousman *et al.* (2016)	✓	✓	✓				✓		✓							
Footer *et al.* (2017)	✓				✓											
Kvach *et al.* (2017)	✓		✓			✓										
Mutale *et al.* (2017)	✓		✓	✓												
Szabo *et al.* (2017)	✓	✓	✓													
Aagaard *et al.* (2018)	✓	✓	✓			✓								×		
Bates *et al.* (2018)	✓			✓												
Cleary *et al.* (2018)	✓		✓	✓	✓											
Doherty *et al.* (2018)	✓	✓		✓	✓					✓			✓	×		
Dzudie *et al.* (2018)	✓															
Foster *et al.* 2018)	✓	✓			✓						✓	✓				
Gross *et al.* (2018)	✓	✓	✓		✓			✓						×		
Muhimpundu *et al.* (2019)	✓			✓												
Spies *et al.* (2018)	✓															
Total	27	16	15	10	7	3	3	3	3	2	1	1	1	6	3	1

There was less variation in the teaching and learning methods used between the different LDPs than there was with the learning content; a small number of teaching and learning methods were found consistently across the reviewed studies. All LDPs (100%) used lectures or workshops as part of the training. The majority (59%) also used real-world project work as the key pedagogic format, an approach which involved asking participants to put their learning into practice through a discrete project, either individually or in groups. These often took the form of quality improvement projects within the workplace. In-person mentoring, coaching or supervision (56%) was the third common approach taken.

Relatively few studies of LDPs (30%) included reference to participants being assessed—most commonly through an individual or group presentation (22%).

### Evaluation of the LDPs

It is important to distinguish the programme evaluation from the individual participant assessment described above. In some instances, these did overlap; for example, Dovey (2002) analysed the project reports used to assess participants of the LDPs to then evaluate the effectiveness of the overall programme.

Twenty-three studies (85%) evaluated the reported LDPs and four (15%) did not. Those studies that included an evaluation component reported 15 different methodologies for evaluation, with most studies using more than one method (ranging between 1 and 8, with the mean being 2.6). These are summarized in Table 4.

**Table 4:** Evaluation methods of the leadership development programmes, analysed across by Kirkpatrick framework categories

	Participant feedback surveys	Participant interviews/FGs	Participant reflections	Pre-post knowledge/ attitude tests	Participant oral exam/assessment	Participant skills logbook	Interviews/FGs with colleagues/community	Participant observation	Project evaluations	Health system or outcome change	Participant career follow-up	Document review	Independent programme evaluation	Reflective discussion between researchers	Attendance records
	Reaction			Learning			Behaviour		Results						
Dovey (2002)			✓						✓				✓		
Perry (2008)								✓					✓		
Kebede *et al.* (2010, 2012)										✓					
Matovu *et al.* (2011)			✓								✓				
Seims *et al.* (2012)										✓					
Abdulmalik *et al.* (2014)															
Kwamie *et al.* (2014)		✓					✓	✓				✓			
Nakanjako *et al.* (2015)			✓								✓	✓			
Wilson *et al.* (2015)	✓		✓					✓				✓	✓	✓	
Downing *et al.* (2016)															
Edwards *et al.* (2015)	✓			✓											
Goldstone and Ntuli, (2016)	✓	✓		✓			✓	✓	✓	✓		✓			
Najjuma *et al.* (2016)	✓	✓													
Ousman *et al.* (2016)	✓		✓	✓		✓									
Footer *et al.* (2017)	✓														
Kvach *et al.* (2017)		✓					✓								
Mutale *et al.* (2017)		✓		✓			✓			✓					
Szabo *et al.* (2017)	✓								✓						
Aagaard *et al.* (2018)	✓	✓		✓					✓						✓
Bates *et al.* (2018)			✓												
Cleary *et al.* (2018)		✓						✓	✓			✓	✓	✓	
Doherty *et al.* (2018)	✓	✓	✓				✓		✓		✓				
Dzudie *et al.* (2018)															
Foster *et al.* 2018)		✓			✓		✓		✓						
Gross *et al.* (2018)	✓														
Muhimpundu *et al.* (2019)															
Spies *et al.* (2018)			✓	✓											
Total	10	9	8	6	1	1	6	5	7	4	**3**	**5**	**4**	**2**	**1**

The wide diversity of methods used, coupled in a number of cases with poor quality of evaluation or poor alignment between the methods and the stated objectives of the LDPs, made it challenging to summarize the results of the evaluations in a coherent way or to draw out useful conclusions. It did, however, highlight the need to consider what methods are being used to evaluate LDPs and how appropriate they are for this purpose, to inform the design of evaluations for future LDPs and priorities for further research. The focus of the next section is therefore on evaluation methods rather than the evaluation findings, and this issue is expanded further in the Discussion.

We used the well-established Kirkpatrick framework for evaluating complex educational and training interventions and programmes to cluster the evaluation methodologies we found across studies. We thus coded evaluation data into the four Kirkpatrick categories of ‘Level 1: reaction’ (how participants felt about the programme), ‘Level 2: learning’ (increases in participants’ knowledge), ‘Level 3: behaviour’ (evidence of participants’ applied learning and skill acquisition) and ‘Level 4: results’ (effects on client or patient outcomes) (Kirkpatrick and Kirkpatrick, 2006).

Studies assessed most commonly learners’ ‘reaction’. Ten studies (37%) used feedback surveys (either online or by paper) to document how participants felt about the programme or their perceptions about what they had learnt. These often combined a quantitative element (e.g. Likert scales) and a qualitative component (such as open comment boxes). Nine studies (33%) conducted interviews and/or focus groups with participants and eight studies (30%) collected participant reflections on the LDP (such as through participant journals, email correspondence or reflective team project reports). ‘Learning’ was mostly assessed through pre/post knowledge or attitude tests (22%). Learners’ ‘behaviour’ was evaluated through participant observation (19%) and interviews or focus groups with colleagues, supervisors or community members (22%). Finally, ‘results’ included evidence of a change in health system outcomes or health outcomes (15%), participant project evaluations (26%) and participant career follow-up (11%).

A few reported methodologies did not fit clearly into any of the Kirkpatrick categories—mostly document review, such as curricula or learning materials (19%), and external programme evaluations (15%).

### Lessons learnt from the LDPs

Many of the studies also identified lessons learnt from the experience of running the LDPs or from their formal evaluation. These were phrased in different ways, including as barriers or facilitators to successful programmes, or suggestions for the future. We rephrased these so that they were all presented as lessons learnt and then grouped them into items. Initial themes and categories were guided by the research question and identified inductively by OJ and iteratively reviewed by KB, AHK and NS until consensus was reached. The three major categories were: design of the LDPs; external engagement and health system integration, and institutionalization and sustainability.

The lessons learnt about the design of the LDPs describe issues around the course structure, curriculum, teaching and learning methods, faculty and student selection. These are listed in Table 5, with the numbers in the second column denoting how many studies these lessons were referenced in.

**Table 5:** Lessons learnt about the design of the LDPs

**Programme design**		
Ensure that the programme is accredited	3	Kebede *et al.* (2012), Goldstone and Ntuli (2016), Mutale *et al.* (2017)
Sustain the follow-up for longer	2	Kwamie *et al.* (2014), Kvach *et al.* (2017)
Ensure consistent administrative support	2	Goldstone and Ntuli (2016), Footer *et al.* (2017)
Coordinate effectively across countries	1	Abdulmalik *et al.* (2014)
Lengthen the intervention to include multiple cycles	1	Kwamie *et al.* (2014)
Provide remote follow-up after the training	1	Muhimpundu *et al.* (2019)
Remain flexible to external changes	1	Kebede *et al.* (2010)
Select data for evaluations carefully	1	Edwards *et al.* (2015)
**Learning content**		
Adapt the curriculum to the specific context	2	Goldstone and Ntuli (2016), Kvach *et al.* (2017)
Ensure a balance between technical skills and critical thinking	1	Kebede *et al.* (2010)
Include emotional intelligence, dealing with any buried personal trauma and stress management	1	Wilson *et al.* (2015)
Include policy and advocacy	1	Kebede *et al.* (2010)
Include systems thinking and reflective practice	1	Kwamie *et al.* (2014)
Provide support for research ethics applications	1	Aagaard *et al.* (2018)
Use a standard curriculum	1	Muhimpundu *et al.* (2019)
**Teaching and learning methods**		
Use real-world case studies	5	Goldstone and Ntuli (2016), Ousman *et al.* (2016), Mutale *et al.* (2017), Doherty *et al.* (2018), Muhimpundu *et al.* (2019)
Include work-based learning	3	Dovey (2002), Perry (2008), Matovu *et al.* (2011)
Use peer learning	3	Goldstone and Ntuli (2016), Ousman *et al.* (2016), Doherty *et al.* (2018)
Close knowledge gaps early to set a common baseline between participants	2	Ousman *et al.* (2016), Muhimpundu *et al.* (2019)
Draw on participant issues in discussions	2	Vélez and Foster (2016), Cleary *et al.* (2018)
Use reflective sessions	2	Ousman *et al.* (2016), Cleary *et al.* (2018)
Use team-based learning	2	Mutale *et al.* (2017), Cleary *et al.* (2018)
Use coaching	1	Dovey (2002)
Use peer facilitators to weaken existing hierarchies	1	Kwamie *et al.* (2014)
**Participants and selection**		
Invite participants from across hierarchies	2	Mutale *et al.* (2017), Cleary *et al.* (2018)
Make the significant workload explicit	1	Doherty *et al.* (2018)
Open the programme to wide range of health professions	1	Nakanjako *et al.* (2015)
Remunerate participants	1	Edwards *et al.* (2015)
Select students carefully	1	Doherty *et al.* (2018)
**Faculty and staff**		
Ensure adequate faculty numbers and availability	2	Matovu *et al.* (2011); Footer *et al.* (2017)
Allocate floating mentors to support where mentors in host institutions become too busy	1	Matovu *et al.* (2011)
Ensure that the programme director is directly engaged in teaching	1	Footer *et al.* (2017)

The second category of external engagement and health system integration of LDPs (see Table 6) describes lessons learnt around how the LDPs fit into the broader context of the education and health systems, including how to address the challenge of participants undertaking a training programme while continuing their day jobs in the health sector.

**Table 6:** Lessons learnt about the external engagement and health system integration of the LDPs

**Engagement and communication**		
Align different expectations of the LDP from diverse stakeholders	2	Kebede *et al.* (2010), Matovu *et al.* (2011)
Ensure that employers are engaged and supportive of the LDP	2	Edwards *et al.* (2015), Doherty *et al.* (2018)
Ensure that senior institutional leadership is supportive of the LDP	2	Goldstone and Ntuli (2016), Aagaard *et al.* (2018)
Engage and communicate the LDP widely across the sector	1	Matovu *et al.* (2011)
Hold regular meetings between academic and host faculty to ensure harmony	1	Matovu *et al.* (2011)
**Embedding in health system**		
Ensure that participants who are also working have protected time for the LDP	4	Matovu *et al.* (2011), Mutale *et al.* (2017), Aagaard *et al.* (2018), Doherty *et al.* (2018)
Provide grants to implement health system projects linked to the LDP	3	(Aagaard *et al.*, 2018; Edwards *et al.*, 2015; Perry, 2008)
Link LDP to government policies	2	Kebede *et al.* (2012), Goldstone and Ntuli (2016)
Ensure that LDP is aligned with the broader health system administrative and governance processes	2	Kebede *et al.* (2012), Cleary *et al.* (2018)
Link LDP to promotions and continuing professional development systems	1	Foster *et al.* (2018)
Link the LDP to creation of new roles in the health system	1	Kebede *et al.* (2012)
Ensure that roles are available for LDP graduates	1	Matovu *et al.* (2011)
Embed the LDP in health system structures	1	Edwards *et al.* (2015)
Ensure that a critical mass of LDP participants are engaged from within the health system	1	Edwards *et al.* (2015)
Support graduates to implement their learning in the workplace	1	Doherty *et al.* (2018)
Enable districts to volunteer to participate in the LDP	1	Kwamie *et al.* (2014)

The final category of lessons learnt relates to how the LDPs can be made sustainable over the long term and embedded into domestic institutions (see Table 7). This was of particular relevance, as so many of the LDPs were funded by donors and delivered by international NGOs.

**Table 7:** Lessons learnt about the sustainability and institutionalization of the LDPs

Ensure self-sufficiency with domestic funding	6	Abdulmalik *et al.* (2014), Kwamie *et al.* (2014), Nakanjako *et al.* (2015), Footer *et al.* (2017), Mutale *et al.* (2017)
Deliver through national or regional institutions	4	Kebede *et al.* (2010), Goldstone and Ntuli (2016), Mutale *et al.* (2017), Foster *et al.* (2018)
Draw on national or regional faculty	4	Matovu *et al.* (2011), Abdulmalik *et al.* (2014), Goldstone and Ntuli (2016), Foster *et al.* (2018)
Ensure country ownership	2	Goldstone and Ntuli (2016), Foster *et al.* (2018)
Train participants to become future mentors and faculty	2	Goldstone and Ntuli (2016), Muhimpundu *et al.* (2019)
Anticipate resource constraints in the setting	1	Kebede *et al.* (2010)

## Discussion

To the best of our knowledge, this is the first review of published literature about LDPs in health care that has a specific focus on Sub-Saharan Africa. This review provides a summary of existing LDPs, including their structure, content and learning methods. Furthermore, it sets out the methods that have been used to evaluate LDPs in Sub-Saharan Africa and maps out the available literature, including its gaps.

The review reveals how rapidly leadership has emerged as a topic of significant interest in health care in Sub-Saharan Africa over recent years. Despite recent advances, the review also indicates the need for more and higher quality research on this subject. We identified several major themes concerning the relevance, design, evaluation and institutionalization of LDPs in Sub-Saharan Africa. We summarize and reflect on these and their implications for LDPs in the Sub-Saharan African context in the sections that follow.

### Broad relevance of leadership development for health professionals in Sub-Saharan Africa

Perhaps the most significant finding of this review is that a substantial number of LDPs have been introduced for health professionals in Sub-Saharan Africa over the last decade, covering a majority of countries in the region. These targeted a wide range of different professional cadres at all stages of the career pathway, from the undergraduate level to senior roles. This suggests that leadership is a common challenge for these health systems and that the introduction of LDPs has widespread relevance in these contexts.

Multiple different formats for the LDPs were successfully implemented, with vastly different class sizes and lengths of duration. Differing approaches to delivering LDPs may, therefore, all be effective, although there is a lack of evidence about whether some formats may have greater impact than others.

### Absence or diversity of theory underpinning the LDPs

A second theme identified by the authors was the absence of clear conceptual or theoretical frameworks underpinning many of the LDPs and their associated studies. In addition, there was considerable theoretical diversity amongst those that did have a clear and explicit concept of leadership or leadership framework. This was further reflected in the inconsistency in the learning content of the LDPs.

It therefore appears that there is currently a lack of consensus on how leadership functions in this specific context .This fundamental disagreement over the purposes and principles of leadership in LDPs raises a question of internal consistency: whether, in other words, all of the authors of these studies were even referring to the same phenomenon when they referred to ‘leadership’, or whether shared language was being used to describe fundamentally different concepts or approaches.

This review highlights the need for more clear and explicit theoretical frameworks, which are critically important to ensure that the various components of the LDPs, such as the learning content and the teaching and learning methods, are aligned towards the particular goal. In addition, it is an essential pre-condition for effective evaluation, because you cannot assess the impact of a programme without being clear what it is intending to achieve and how.

### Overlap between leadership and management concepts

A related conceptual factor that emerged from many of the studies was the apparent, albeit rather implicit, conceptual overlap between leadership and management. This was evidenced most clearly in the review of the LDP content, with ‘project management’ included as a topic in 13 of the LDPs (48%), making it the second most common topic overall. Indeed, five of the studies (19%) specifically referred to ‘leadership and management’ in their title (Perry, 2008; Matovu *et al.*, 2011; Seims *et al.*, 2012; Mutale *et al.*, 2017; Foster *et al.*, 2018).

This intersection has been discussed extensively in the literature, with Fernandez *et al.* (2010) noting that ‘The distinction between management and leadership remains a conceptual knot that is difficult to untangle’. In many ways, it would seem sensible for training programmes for health professionals to cover both concepts as they will likely be responsible for overseeing both change and performance at the same time. Indeed, Bolden *et al.* (2011) suggest that, ‘for maximum effect we should seek to recruit and develop “leader–managers” capable of adopting the role in its most holistic form’. Therefore, splitting hairs over definitions of the two, or trying to separate them into different training programmes, may not only be unnecessary, but also counterproductive.

There is, however, clearly a need to get the balance right between leadership and management, and an assessment of the content of the LDPs does raise questions about whether the leadership aspects were given sufficient attention in all cases. For example, in Frenk’s (2010, p. 2) call for better leadership in health care that is quoted in the introduction, he emphasizes the importance of ‘strategic vision, technical knowledge, political skills and ethical orientation’. However, this review found that topics such as ‘strategy development’, ‘self-reflection’ and ‘network building’ were only included in a small minority of the LDPs. Perhaps even more notable was how rarely ‘ethics and professional values’ were referenced, appearing in just three (11%) of the studies. This may be a particularly important omission, given Bennis and Nanus’s description of how ‘Managers are people who do things right and leaders are people who do the right thing’ [1985, p.21, in Fernandez *et al.* (2010*)*].

The rapid increase in published papers on leadership in this setting, documented in this review, suggests that the term ‘leadership’ has become very popular in recent years. That opens the question of whether the label of ‘leadership’ has been applied to some programmes that are largely focused on management training, perhaps for funding or branding purposes.

### Translation of high-income country leadership models in Sub-Saharan Africa

Three of the studies (11%) explicitly drew on leadership frameworks that were developed outside of the region, in particular from North America and in Europe (Mutale *et al.*, 2017; Cleary *et al.*, 2018; Muhimpundu *et al.*, 2019). In addition, many others were designed or delivered in significant part by faculty from outside the region, and it seems likely that they will have brought models or approaches of leadership with them from those contexts.

The universality of leadership concepts and frameworks for health professionals remains an open question. Further research is needed into whether there are distinctive features to the leadership roles, the health system context and the wider societal cultures within Sub-Saharan Africa that need to be better understood and built around. These might be broadly relevant across the region or unique for particular countries or other contexts. Although the literature on this is limited, the GLOBE study on culture, leadership and organizations did identify distinctive characteristics for leadership and the leadership context in different regions around the world, including in Sub-Saharan Africa (House *et al*., 2004) . Thus, rather than replicating North American or European leadership models, more work should be done to develop specific leadership frameworks within the region, as was referenced by studies from Zambia and South Africa (Goldstone and Ntuli, 2016; Foster *et al.*, 2018).

### Challenges with the evaluation approaches used by the LDPs

The wide-ranging diversity in the evaluation methods used demonstrates a gap in having clear standards or agreed approaches to evaluating LDPs in this context. Three key reflections emerge from analysing the studies, and specifically why the evaluation was so complex and why gaps in the evaluation approach were found.

The first relates to timing. Leadership development is often considered to be part of a long-term process of personal and professional development for participants, the effects of which may not be seen until many years in the future as they move into more senior job roles or face unforeseen challenges (Day, 2000). The programme evaluation methods were almost always short term, however, with a lack of funding or time to follow-up participants over many years. The short timeframes that were often adopted go some way to explaining why so much of the evaluation in the studies focused on immediate measures, such as reaction and learning, rather than on evidence of changes in behaviour or results.

Linked to this is the issue of causation, the fact that leadership development is an upstream intervention that occurs within the enormous complexity of a health system. The effects of better leadership practice by a health professional will almost never directly impact on patient care but will, as shown in the theories of change of the LDPs, work through numerous intermediate steps, such as changes to staff motivation or more effective use of resources. These intermediate steps are also influenced by multiple other variables, such as changes to workloads or budget allocations. This complexity makes it inherently difficult to attribute any improvement in health outcomes to an LDP. This is compounded by the earlier issue of most LDPs not being theory-driven, resulting in a lack of clarity on what the intermediate steps are expected to be. Future LDPs could strengthen their evaluation by basing it on a more explicit theory of change that identifies how the training is expected to make a difference to the participants and the health system more broadly.

Thirdly, the evaluation was mostly applied research that was driven by the priorities of programme delivery rather than evidence generation. Of the 25 that declared the sources of funding for the study, 20 (74%) were funded either by aid agencies [such as The United States Agency for International Development (USAID)] or philanthropic or corporate social responsibility grants (such as Sanofi). Only five studies (19%) stated that they were funded by research agencies, such as the National Institutes of Health (NIH) in the USA (Kwamie *et al.*, 2014; Edwards *et al.*, 2015; Najjuma *et al.*, 2016; Dzudie *et al.*, 2018; Spies *et al.*, 2018). The relative lack of independent research funding for evaluation of the LDPs not only raises questions about the objectivity of the evaluations but also suggests that issues of research design are likely to have been secondary to programme design in most instances. Where possible, future LDPs should seek to get independent funding to evaluate their programmes and research funders should prioritize this area.

Alongside these broader issues with the evaluation, there are some specific concerns about the validity of some of the most commonly used evaluation methods of the LDPs.

Previous research on using self-assessment of learning or ability by participants, the most common evaluation method identified in the review, raises questions about the efficacy of this approach to evaluating learning. In their seminal study on self-assessments, Kruger and Dunning found that ‘the incompetent will tend to grossly overestimate their skills and abilities’, since the knowledge required to “produce” correct judgement is the same as that required to “recognize” correct judgement (1999, p. 1122). By contrast, more competent people will tend to underestimate their own abilities. They found that training can therefore have a somewhat counter-intuitive effect of lowering the self-perceived abilities of participants who are least competent, as they start to ‘know what they don’t know’ and are therefore better able to calibrate their own abilities.

Similar findings were made by Sitzman *et al.* in their meta-analysis assessing the validity of using self-assessments of knowledge in workplace training, who found only a moderate correlation with participants’ cognitive learning, with a stronger correlation with motivation and satisfaction (2010). They go on to recommend a more limited role for self-assessment in evaluating learning. One study in this review, Edwards *et al.*, indeed finds this phenomenon in their evaluation, noting that ‘author experience working with (participants) on their evaluation projects suggests that in some cases, their self-reported confidence in using these skills may have outstripped their actual ability’ (2016). While using self-assessment to evaluate LDPs may inform us about participants’ motivation, and satisfaction with the programme, it likely tells us little about increased leadership capability. This method should therefore be combined with other evaluation approaches and, when analysed, the results should be explained as largely demonstrating participant attitudes only.

While pre/post-tests of knowledge or attitudes, used in five of the studies, or a skills logbook, used in one study, may be more objective instruments than self-perceived ability, they are also quite limited in the domains of leadership that they can assess. Furthermore, as with self-assessment of ability, these tests also do not tell us whether the training led to a change of behaviour, or whether any changes in behaviour led to any impacts for the health system.

Of the studies that did look at results, the most commonly used approaches to evaluation were assessing changes in health systems or health outcomes (7%), or the impacts of a specific health project (22%). These methods also have significant limitations, however, as by instructing participants to undertake a project, and often giving them time and resources to implement it, it is unsurprising that the projects had some kind of impact. Such findings do not tell us, however, if the impact seen is because the participants are more effective in their leadership practice than they had been before the LDP or is simply a consequence of them doing a project.

Similarly, while following up participants on their careers (7%) does provide a longer term view on their development, as a proxy measure, it is limited to informing us about the jobs they successfully apply for, and not whether their leadership capabilities really have improved. For example, their career success could be more the result of the qualification they received from the LDP than actual improved leadership capability.

One way to attribute evaluations in the ‘results’ category to improved leadership practice due to the LDP would be to have a control group, which two studies used. Edwards *et al.*’s leadership hubs in Jamaica, Kenya, Uganda and South Africa had three control districts alongside their three intervention districts in each country (except South Africa, which had no control districts). Seims *et al.*’s leadership training for 67 facility and district health teams in Kenya had 59 non-random comparison groups and measured changes in a specific health access of coverage indicator at baseline, end of intervention and 6-months post-intervention. Introducing control groups to operational research may not be possible in all contexts, for example, because policy-makers may require an entire cohort to participate in the intervention, selection bias may occur or participants hard to recruit for the control arm. Good planning and active engagement with policy-makers and participants may allow these potential problems to be overcome, however, and control groups should be considered where possible.

Given these significant limitations, a shortlist of the evaluation methodologies that may be most suitable for assessing LDPs in this context can be suggested. Qualitative participant reflections provide holistic evidence of how thinking and self-reflection are developing and are perhaps more immune to the Kruger–Dunning effect as they are more than just a self-assessment of ability, although they are obviously subjective and do not easily allow for comparison between studies. Participant observation and interviews or focus groups with colleagues or community members are likely to be effective at informing us about changes in participant leadership behaviour, but again, their qualitative nature makes comparison between studies challenging. Finally, evaluation of the results of the LDP, such as through the impact of health projects or changes in career trajectory, could be an effective approach but only if a control group is included.

### Importance of institutionalizing LDPs to ensure effectiveness and sustainability

The final theme, which emerged strongly from the lessons learnt by authors of the studies, was the importance of institutionalizing the LDPs (i.e. embedding them within an organization or system). Many of the lessons pointed towards this issue, from the need for programmes to be accredited, to having manageable workloads, recognition from across the sector, alignment with the health system and resourcing with domestic funding.

Institutionalizing the LDPs therefore appears to be key to both their effectiveness and their sustainability. Yet, as noted in the previous section, a large majority of the LDPs were funded either by philanthropic or by international donor funding, which are both inherently unstable sources of financing over the long term. Similarly, the design and delivery of the LDPs often appeared to be reliant on international NGOs, partnerships or faculty. This often made the LDPs extremely expensive to run, with Nakanjanko *et al.* noting that the price of their programme was up to $40 000 per participant for a year (2015), affecting sustainability and limiting potential scalability.

There were several exceptions to this, however, that provide examples for how LDPs can be institutionalized and sustained for the long term. Najjuma *et al.*’s undergraduate module for medical students was accredited by the Ugandan university and taught by its existing. Similarly, Doherty *et al.*’s Oliver Tambo Fellowship was embedded in South Africa universities, as was Kebede’s Masters in Health Care Administration in Ethiopia, which was also closely aligned to new national health policies and the job descriptions of the hospital CEOs who were participating. A model for best practice might therefore be to ensure that LDPs are led by, and embedded within, national institutions from the outset and accompanied by a clear plan for follow-up, health system integration and sustainability.

## Limitations

This review has limitations. Only 27 studies were identified, and although efforts were made to include articles written in French, no such articles were identified through the search; hence, we suspect that studies may have been missed, particularly from Francophone countries. The search strategy largely focused on papers published in academic journals, and some evaluations developed as reports or in other formats were likely not picked up by the search. Moreover, the definitions or interpretations of leadership used in these studies were varied and sometimes vague. This lack of clarity contributes to the challenges in drawing firm conclusions. Furthermore, inferences were often drawn when information was not explicitly stated in a study, but in some cases, it may be that activities took place that were not reported. Lastly, the evaluation of a number of studies lacked methodological rigour and consistency, making it challenging to draw firm conclusions on the effectiveness of the LDPs or to make comparisons.

One implication of this is that the findings may be less representative of, or relevant to, francophone countries. Furthermore, the lack of rigour in the evaluation of some studies means that this scoping review should be used more to understand the range of approaches and programme designs that have been used by LDPs for health workers in Sub-Saharan African than to determine whether one model or programme design is more effective than another (i.e. we advocate using the findings of the review in a formative rather than in a summative manner).

## Further research

This review identified a number of important areas for further research. Firstly, greater clarity is needed on what effective leadership by health professionals in Sub-Saharan looks like, and whether leadership concepts or models that have been developed in other regions need to be adapted, expanded or re-imagined for this context. That research would inform what best practice should be for the learning content and methods for LDPs. More robust evaluations are then needed, with longer term follow-up, to assess the effectiveness of the LDPs and drive continuous learning.

## Implications for policy and practice

A number of implications for practice and policy relating to strengthening healthcare leadership in Sub-Saharan Africa have been identified in this review. The evidence of significant and growing attention to LDPs over the last few years suggests their importance to health systems strengthening and highlights the need for greater focus and investment in LDPs by policy-makers, training institutions and donors. The review also summarized a range of approaches and options for the design and evaluation of LDPs which, along with the lessons learnt that were identified by the authors of the 27 studies, can be used to inform future programme development.

## Conclusion

This review is the first of its kind to summarize LDPs in health care that has a specific focus on Sub-Saharan Africa. Whereas no ‘one best’ approach emerged from the review, our findings support the need for LDPs to be more theory-based, better integrated into existing systems, accredited and more effectively evaluated. There should also be greater emphasis on institutionalization and financial sustainability from their early development phase—otherwise, they can only be ephemeral. The review findings should be useful to academics, policy-makers and practitioners who are seeking to develop, strengthen or evaluate LDPs for health professionals in the region. Ultimately, we hope to have demonstrated the diversity of approaches that have been adopted to date and shared the key lessons from these experiences to inform future programme development and leadership practice.

## Supplementary data

Supplementary data are available at *Health Policy and Planning* online.

## Supplementary Material

czaa078_Supplementary_Data
