# Supplementary material for: Interventions to strengthen the leadership capabilities of health professionals in Sub-Saharan Africa: a scoping review
Source: Health Policy Plan. 2020 Dec 13;36(1):117–33. doi: 10.1093/heapol/czaa078 (PMC7938510; doi:10.1093/heapol/czaa078)
Supplement: czaa078_Supplementary_Data [file czaa078_supplementary_data.zip › Appendix 1.docx]

**Appendix 1: Definitions of learning content categories for leadership development programmes summarised in Table 3**

| **Content Categories** | **Definition** |
| --- | --- |
| Concepts or Experiences of Leadership | definitions, approaches, theories, principles or attributes of leadership that have been theorised or evaluated; personal reflections on specific leadership experiences in particular contexts |
| Project Management | the practice of initiating, planning, executing, controlling, and closing the work of a team to achieve specific goals and meet specific success criteria at the specified time |
| Change Management or Quality Improvement | the coordination of a structured period of transition in order to achieve lasting change within an organization; an approach to improvement of service systems and processes through the routine use of health and programme data to meet patient and programme needs. |
| Technical, Public Health or Health Systems Topics | Specific technical topics related to clinical, public health or health systems issues (e.g. management of HIV, epidemiology or a summary of health system financing) |
| Policy, Influence & Advocacy | a set of ideas or plans that is used as a basis for making decisions in an organization; the power to make other people agree with your opinions or do what you want; the attempt to influence the decisions of an organization |
| Relationship Management | the understanding of interpersonal connections between people and within a team, and how to navigate these to achieve organisational goals through relational approaches such as collaboration, empathy, trust and empowerment |
| Communication | the process by which messages or information is sent from one place or person to another; the exchange of information and the expression of feeling that can result in understanding |
| Strategy Development | an organisational management activity that is used to set priorities, focus energy and resources, strengthen operations, ensure that employees and other stakeholders are working toward common goals, establish agreement around intended outcomes/results, and assess and adjust the organisation's direction in response to a changing environment. |
| Supervision | the act of watching a person or activity and making certain that everything is done correctly, safely, etc - including supporting their professional development through mentoring, coaching and feedback |
| Self Reflection | the activity of thinking about your own feelings and behaviour, and the reasons that may lie behind them |
| Ethics or Professional Values | the standards and moral conduct that govern the profession and its members |
| Systems Thinking | a holistic approach to analysis that focuses on the way that a system's constituent parts interrelate and how systems work over time and within the context of larger systems. |
| Network Building | meeting to form professional relationships and to recognize, create, or act upon opportunities, share information and seek potential partners for collaborative initiatives |
| Team working | co-operation between those who are working on a task, the willingness of a group of people to work together to achieve a common aim. |
| Workplace Practice | the surrounding conditions in which a team operates, including physical conditions, work processes or procedures and organisational culture |
