# Supplementary material for: Interventions to strengthen the leadership capabilities of health professionals in Sub-Saharan Africa: a scoping review
Source: Health Policy Plan. 2020 Dec 13;36(1):117–33. doi: 10.1093/heapol/czaa078 (PMC7938510; doi:10.1093/heapol/czaa078)
Supplement: czaa078_Supplementary_Data [file czaa078_supplementary_data.zip › Box 1.docx]

**Box 1: Eligibility Criteria & Search String**

**Inclusion Criteria Exclusion Criteria**

- Published on/after 1978
- Describes a previous or existing leadership development intervention
- Targets health professionals as participants
- Includes at least one country in Sub-Saharan Africa
- Published peer-reviewed journal, technical report or academic thesis
- Written in English; or French with English abstract available
- Published before 1978
- Does not include a leadership intervention, or intervention not yet delivered
- Does not specifically target or reference health professionals
- Does not include at least one country in Sub-Saharan Africa
- Conference abstract without full text available
- No English abstract available

**Search String**

(Africa* OR Angola* OR Benin* OR Botswana OR Batswana OR "Burkina Faso" OR Burkinese OR Burundi* OR Cameroon* OR "Cape Verd*" OR "Cabo Verd*" OR "Central African Republic" OR Chad* OR Comoros OR Comoran OR Congo* OR "Democratic Republic of Congo" OR "Equatorial Guinea*" OR Eritrea* OR Ethiopia* OR Gabon* OR Gambia* OR Ghana* OR Guinea* OR "Guinea Bissau" OR "Ivory Coast" OR "Cote d’Ivoire" OR Kenya* OR Lesotho OR Basoth* OR Liberia* OR Madagasca* OR Malawi* OR Mali* OR Mauritania* OR Mauretania* OR Maurit* OR Mayot* OR Mozambiq* OR Mocambiq* OR Namibia* OR Niger* OR Rwanda* OR "Sao Tome" OR Senegal* OR Seychell* OR "Sierra Leone*" OR Somali* OR "South Africa*" OR Sudan* OR Swazi* OR eSwatini OR Tanzania* OR Togo* OR Uganda* OR "Western Sahara*" OR Zaire OR Zambia* OR Zimbabwe*) AND (Health* OR medic* OR clinic* OR hospital* OR "primary care" OR doctor* OR physician* OR nursing OR nurse* OR midwife* OR pharmacist* OR pharmacy OR dental OR dentist* OR psychiatrist* OR psychologist* OR surgeon*) AND (leader*) NOT ("local leader*" or "opinion leader*" OR "peer health leader*" OR "traditional leader*" OR "faith leader*" or "government leader*" OR "community leader*" OR "religious leader*" OR "muslim leader*" OR "christian leader*" OR "african american")
