# Supplementary material for: Interventions to strengthen the leadership capabilities of health professionals in Sub-Saharan Africa: a scoping review
Source: Health Policy Plan. 2020 Dec 13;36(1):117–33. doi: 10.1093/heapol/czaa078 (PMC7938510; doi:10.1093/heapol/czaa078)
Supplement: czaa078_Supplementary_Data [file czaa078_supplementary_data.zip › Box 2.docx]

**Box 2: Frequency Sub-Saharan African Countries were included in studies**

South Africa 8

Uganda 8

Ethiopia 4

Kenya 4

Zambia 3

Botswana 2

Ghana 2

Malawi 2

Mozambique 2

Rwanda 2

Tanzania 2

Zimbabwe 2

Cameroon 1

eSwatini 1

Gambia, The 1

Lesotho 1

Liberia 1

Mauritius 1

Namibia 1

Nigeria 1

Seychelles 1

Sierra Leone 1

South Sudan 1
