# Supplementary material for: Interventions to strengthen the leadership capabilities of health professionals in Sub-Saharan Africa: a scoping review
Source: Health Policy Plan. 2020 Dec 13;36(1):117–33. doi: 10.1093/heapol/czaa078 (PMC7938510; doi:10.1093/heapol/czaa078)
Supplement: czaa078_Supplementary_Data [file czaa078_supplementary_data.zip › Box 3.docx]

**Box 3: Case Studies of Three LDPs from the Scoping Review**

﻿**The Ethiopia Hospital Management Initiative (EHMI)** (Kebede et al., 2010, 2012)

The EHMI was a large-scale initiative established by the Federal Ministry of Health in Ethiopia to strengthen hospital management capacity across the country. It took a comprehensive approach that included the restructuring of senior management roles in government hospitals by creating the post of Chief Executive Officer (CEO), the development of ﻿*Standards for hospital management in Ethiopia* and *the Blueprint for hospital management in Ethiopia* and the introduction of a ﻿2-year executive-education style Masters of Healthcare and Hospital Administration (MHA) programme.

The MHA was targeted at new hospital CEOs and was hosted by Jimma University in Ethiopia with initial support from Yale University in the USA. The course consisted of a series of three-week blocks, every four months, with participants working in their hospitals the rest of their time. There were approximately 25-30 participants in each cohort. The learning content included public health, health policy, ﻿problem solving, supply chain management, hospital operations, health care financial management, strategic management, nursing management, human resource management and leadership development. When back at their hospitals, participants sent weekly progress reports to their faculty and received on-site supervision.

**Multidisciplinary leadership training for undergraduates at Mbarara University of Science and Technology (MUST) in Uganda** (Najjuma et al., 2016)

When the Ugandan Ministry of Health identified the need to strengthen the leadership and management skills of health professionals in the country, MUST decided to introduce a leadership training programme for all undergraduates studying ﻿a Bachelor’s degree in nursing, medicine and surgery, pharmacy, and medical laboratory science.

The approximately 250 undergraduate health sciences students were given one week of teaching in leadership theory before being allocated to multidisciplinary teams of 7-10 people. Each team was then placed in a rural community for five weeks, with the objective of working with that community to identify a health-related problem where they could intervene.

**Wellness for Effective Leadership (WEL) in South Africa** (Wilson et al., 2015)

The WEL programme was undertaken by over 400 frontline managers in the public sector in South Africa from 2009 to 2014, aimed at building emotional intelligence and developing personal and interpersonal competencies. It was based on the premise that “﻿at the core of transformation of any service are the individuals who run the services, and that the changes brought about through greater self-awareness and self-care, perceptions of well-being and reduced stress, lead to an increased ability to manage stressful situations and conflicts.”

The standard programme consisted of three two-day workshops, followed by a final one-day workshop, with a six to eight-week gap between each to allow for reflection and experimentation. The workshops began with participants reflecting on their own life’s journey and contexts, and included a screening for ﻿compassion satisfaction, risk of burnout and secondary traumatic stress. The second and third workshop were then tailored to address the major issues that had been identified, before a final workshop, where participants would report-back to colleagues, family members and visitors.
