# Supplementary material for: Interventions to strengthen the leadership capabilities of health professionals in Sub-Saharan Africa: a scoping review
Source: Health Policy Plan. 2020 Dec 13;36(1):117–33. doi: 10.1093/heapol/czaa078 (PMC7938510; doi:10.1093/heapol/czaa078)
Supplement: czaa078_Supplementary_Data [file czaa078_supplementary_data.zip › Box 4.docx]

**Box 4: Definitions of Conceptual Approaches to Leadership**

**Referenced in the Studies**

**Individualised** *﻿Effective performance by an individual, group, or organization is assumed to depend on leadership by an individual with the skills to find the right path and motivate others to take it. (Yukl, 1999, p. 292)*

**Pluralised** *Leadership as an emergent network of relations, which is a shared phenomenon, encompassing several leaders who may be both formally appointed and emerge more informally. (White, Currie, & Lockett, 2016, p. 280)*

**Distributed/** *Sub-categories of pluralised leadership where leadership*

**Collective** *functions may be shared by several members of a group, allocated to individual members or performed by different people at different times. (Yukl, 1999, p. 292)*

**Transformational** *The process whereby a person engages with others and creates a connection that raises the level of motivation and morality in both the leader and the follower. (Northouse, 2015, p. 164)*

**Relational** *Leadership as an interpersonal phenomenon associated with collaboration, empathy, trust and empowerment (Cummings et al., 2010 in Cleary, Du Toit, Scott, & Gilson, 2018)*
