# Supplementary material for: Interventions to strengthen the leadership capabilities of health professionals in Sub-Saharan Africa: a scoping review
Source: Health Policy Plan. 2020 Dec 13;36(1):117–33. doi: 10.1093/heapol/czaa078 (PMC7938510; doi:10.1093/heapol/czaa078)
Supplement: czaa078_Supplementary_Data [file czaa078_supplementary_data.zip › Table 1.docx]

**Table 1: Summary of the studies and the structure of the leadership development programmes, listed in order of publication year**

| **Author** | **Country** | **Context** | **Target Group of Participants** | **Number of Participants** | **Length** | **Qualification** |
| --- | --- | --- | --- | --- | --- | --- |
| Dovey (2002) | South Africa | On-the-job programme | Interdisciplinary  (District health management teams) | 12-20 in each team | 2 years | Certificate in District Health Management |
| Perry (2008) | Mozambique | On-the-job programme | Interdisciplinary  (District health management teams) | Approx 25 | 2 years |  |
| Kebede et al (2010 & 2012) | Ethiopia | On-the-job programme | Medical doctors  (newly-appointed CEOs) | 24 per cohort | 2 years | Masters of Hospital and Health Care Administration |
| Matovu et al (2011) | Uganda | Training programme followed by placement | Interdisciplinary  (HIV practitioners from clinical and management backgrounds) | 77 fellows over 8 years | 2 years |  |
| Seims et al (2012) | Kenya | On-the-job programme | Interdisciplinary  (District or facility managers including medical doctors, nurses and administrators) | 67 teams | 6 months |  |
| Abdulmalik et al (2014) | ﻿The Gambia, Ghana, Liberia, Nigeria, Sierra Leone | Short-course | Interdisciplinary  (Mental health practitioners including clinicians and reps from government and civil society) | 106 over 4 cohorts (21 per cohort) | 2 weeks |  |
| Kwamie et al (2014) | Ghana | On-the-job programme | Interdisciplinary  (District and facility managers) | 4-7 per team | 6 months |  |
| Nakanjako et al (2015) | Uganda | Training programme followed by placement | Interdisciplinary  (Nurses and medical doctors) | 14 over 4 years (4 per cohort) | 1 year |  |
| Wilson et al (2015) | South Africa | On-the-job programme | Interdisciplinary  (District, Subdistrict and facility managers) | 400 over 6 years | 6 months |  |
| Downing et al (2016) | Uganda | On-the-job programme | Nurses  (Palliative care nurses) | 20 per cohort | 2 years |  |
| Edwards et al (2016) | Jamaica, Kenya, Uganda, South Africa | On-the-job programme | Interdisciplinary  (HIV nurses, researchers and reps of government and civil society) | 167 in 12 hubs | 5 years |  |
| Goldstone et al (2016) | South Africa | Fellowship with part-time postgraduate degree course | Interdisciplinary  (Provincial, District and facility managers) | 178 fellows over 4 years (94 enrolled PGDip, 75 enrolled MPH) | 1-2 years | Postgraduate Diploma, or  Masters in Public Health |
| Najjuma et al (2016) | Uganda | Training programme followed by placement | Interdisciplinary  (Undergraduate health sciences students) | 242 per cohort | 6 weeks | Undergraduate module |
| Ousman et al (2016) | Botswana, Kenya, Uganda, Tanzania, USA | Training programme followed by placement | Interdisciplinary  (HIV professionals including medical doctors, nurses, public health specialists and pharmacists) | 100 over 6 years | 1 year |  |
| Footer et al (2017) | Ethiopia | On-the-job programme | Physiotherapists | 17 per cohort | 4 years | Doctor of Physiotherapy (DPH) |
| Kvach et al (2017) | Ethiopia | International Fellowship | Interdisciplinarty  (female Health Sciences Faculty) | 9 per cohort | 2 weeks |  |
| Mutale  et al (2017) | Zambia | On-the-job programme | Interdisciplinary  (District health managers) | 767 over 2 years | 6-12 months | Diploma in Management & Leadership |
| Szabo et al (2017) | South Africa | On-the-job programme. | Interdisciplinary  (mental health practitioners) | 15 | 2 years |  |
| Aagaard et al (2018) | Zimbabwe | On-the-job programme | Interdisciplinary  (Health Sciences Faculty) | 42 over 3 cohorts (14 per cohort) | 1 year |  |
| Bates et al (2018) | Malawi | Short-course | Interdisciplinary  (from diverse, health-related settings) | 21 per cohort | 5 days |  |
| Cleary et al (2018) | South Africa | On-the-job programme | Interdisciplinary  (District and facility managers, mostly from nursing backgrounds) | 15 per cohort | 4 years |  |
| Doherty et al (2018) | South Africa | On-the-job programme | Interdisciplinary  (senior public health managers) | 91 over 6 cohorts (15 per cohort) | 18 months | Postgraduate Diploma in Health Management |
| Dzudie et al (2018) | Cameroon | Conference | Interdisciplinary  (HIV clinicians and researcher) |  | 1 day |  |
| Foster et al 2018) | Zambia | On-the-job programme | Nurses  (District and facility managers) | 23 per cohort | 1 year | Certificate in Leadership & Management Practice |
| Gross et al (2018) | ﻿17 Countries in SSA | On-the-job programme | Nurses & Midwives  (from ministry, academia and associations) | 4 per country | 3-4 years |  |
| Muhimpundu et al (2018) | Rwanda | Short-course | Interdisciplinary  (NCD programme managers) | 14 in the cohort | 4 days |  |
| Spies et al (2018) | Uganda | Short-course | Nurses | 18 | 2 days |  |
