# Supplementary material for: Interventions to strengthen the leadership capabilities of health professionals in Sub-Saharan Africa: a scoping review
Source: Health Policy Plan. 2020 Dec 13;36(1):117–33. doi: 10.1093/heapol/czaa078 (PMC7938510; doi:10.1093/heapol/czaa078)
Supplement: czaa078_Supplementary_Data [file czaa078_supplementary_data.zip › Table 5.docx]

**Table 5: Lessons Learned about the Design of the LDPs**

| **Programme Design** | | |
| --- | --- | --- |
| Ensure the programme is accredited | 3 | (Goldstone & Ntuli, 2016; Kebede et al., 2012; Mutale et al., 2017) |
| Sustain the follow-up for longer | 2 | (Kvach et al., 2017; Kwamie et al., 2014) |
| Ensure consistent administrative support | 2 | (Footer et al., 2017; Goldstone & Ntuli, 2016) |
| Coordinate effectively across countries | 1 | (Abdulmalik et al., 2014) |
| Lengthen the intervention to include multiple cycles | 1 | (Kwamie et al., 2014) |
| Provide remote follow-up after the training | 1 | (Muhimpundu et al., 2018) |
| Remain flexible to external changes | 1 | (Kebede et al., 2010) |
| Select data for evaluations carefully | 1 | (Edwards et al., 2016) |
|  | | |
| **Learning Content** | | |
| Adapt the curriculum to the specific context | 2 | (Goldstone & Ntuli, 2016; Kvach et al., 2017) |
| Ensure a balance between technical skills and critical thinking | 1 | (Kebede et al., 2010) |
| Include emotional intelligence, dealing with any buried personal trauma and stress management | 1 | (Wilson et al., 2015) |
| Include policy and advocacy | 1 | (Kebede et al., 2010) |
| Include systems thinking and reflective practice | 1 | (Kwamie et al., 2014) |
| Provide support for research ethics applications | 1 | (Aagaard et al., 2018) |
| Use a standard curriculum | 1 | (Muhimpundu et al., 2018) |
|  | | |
| **Teaching & Learning Methods** | | |
| Use real-world case studies | 5 | (Doherty et al., 2018; Goldstone & Ntuli, 2016; Muhimpundu et al., 2018; Mutale et al., 2017; Ousman et al., 2016) |
| Include work-based learning | 3 | (Dovey, 2002; Matovu et al., 2011; Perry, 2008) |
| Use peer learning | 3 | (Doherty et al., 2018; Goldstone & Ntuli, 2016; Ousman et al., 2016) |
| Close knowledge gaps early to set a common baseline between participants | 2 | (Muhimpundu et al., 2018; Ousman et al., 2016) |
| Draw on participant issues in discussions | 2 | (Cleary et al., 2018; Velez & Foster, 2016) |
| Use reflective sessions | 2 | (Cleary et al., 2018; Ousman et al., 2016) |
| Use team-based learning | 2 | (Cleary et al., 2018; Mutale et al., 2017) |
| Use coaching | 1 | (Dovey, 2002) |
| Use peer facilitators to weaken existing hierarchies | 1 | (Kwamie et al., 2014) |
|  | | |
| **Participants & Selection** | | |
| Invite participants from across hierarchies | 2 | (Cleary et al., 2018; Mutale et al., 2017) |
| Make the significant workload explicit | 1 | (Doherty et al., 2018) |
| Open the programme to wide range of health professions | 1 | (Nakanjako et al., 2015) |
| Remunerate participants | 1 | (Edwards et al., 2016) |
| Select students carefully | 1 | (Doherty et al., 2018) |
|  | | |
| **Faculty & Staff** | | |
| Ensure adequate faculty numbers & availability | 2 | (Footer et al., 2017; Matovu et al., 2011) |
| Allocate floating mentors to support where mentors in host institutions become too busy | 1 | (Matovu et al., 2011) |
| Ensure the programme director is directly engaged in teaching | 1 | (Footer et al., 2017) |
