# Supplementary material for: Interventions to strengthen the leadership capabilities of health professionals in Sub-Saharan Africa: a scoping review
Source: Health Policy Plan. 2020 Dec 13;36(1):117–33. doi: 10.1093/heapol/czaa078 (PMC7938510; doi:10.1093/heapol/czaa078)
Supplement: czaa078_Supplementary_Data [file czaa078_supplementary_data.zip › Table 6.docx]

**Table 6: Lessons Learned about the External Engagement and Health System Intergration of the LDPs**

| **Engagement & Communication** | | |
| --- | --- | --- |
| Align different expectations of the LDP from diverse stakeholders | 2 | (Kebede et al., 2010; Matovu et al., 2011) |
| Ensure employers are engaged and supportive of the LDP | 2 | (Doherty et al., 2018; Edwards et al., 2016) |
| Ensure senior institutional leadership is supportive of the LDP | 2 | (Aagaard et al., 2018; Goldstone & Ntuli, 2016) |
| Engage and communicate the LDP widely across the sector | 1 | (Matovu et al., 2011) |
| Hold regular meetings between academic and host faculty to ensure harmony | 1 | (Matovu et al., 2011) |
|  | | |
| **Embedding in Health System** | | |
| Ensure participants who are also working have protected time for the LDP | 4 | (Aagaard et al., 2018; Doherty et al., 2018; Matovu et al., 2011; Mutale et al., 2017) |
| Provide grants to implement health system projects linked to the LDP | 3 | (Aagaard et al., 2018; Edwards et al., 2016; Perry, 2008) |
| Link LDP to government policies | 2 | (Goldstone & Ntuli, 2016; Kebede et al., 2012) |
| Ensure LDP is aligned with the broader health system administrative and governance processes | 2 | (Cleary et al., 2018; Kebede et al., 2012) |
| Link LDP to promotions and continuing professional development systems | 1 | (Foster et al., 2018) |
| Link the LDP to creation of new roles in the health system | 1 | (Kebede et al., 2012) |
| Ensure roles are available for LDP graduates | 1 | (Matovu et al., 2011) |
| Embed the LDP in health system structures | 1 | (Edwards et al., 2016) |
| Ensure a critical mass of LDP participants are engaged from within the health system | 1 | (Edwards et al., 2016) |
| Support graduates to implement their learning in the workplace | 1 | (Doherty et al., 2018) |
| Enable districts to volunteer to participate in the LDP | 1 | (Kwamie et al., 2014) |
