# Supplementary material for: Interventions to strengthen the leadership capabilities of health professionals in Sub-Saharan Africa: a scoping review
Source: Health Policy Plan. 2020 Dec 13;36(1):117–33. doi: 10.1093/heapol/czaa078 (PMC7938510; doi:10.1093/heapol/czaa078)
Supplement: czaa078_Supplementary_Data [file czaa078_supplementary_data.zip › Table 7.docx]

**Table 7: Lessons Learned about the Sustainability and Institutionalisation of the LDPs**

| Ensure self-sufficiency with domestic funding | 6 | (Abdulmalik et al., 2014; Footer et al., 2017; Kwamie et al., 2014; Mutale et al., 2017; Nakanjako et al., 2015) |
| --- | --- | --- |
| Deliver through national or regional institutions | 4 | (Foster et al., 2018; Goldstone & Ntuli, 2016; Kebede et al., 2010; Mutale et al., 2017) |
| Draw on national or regional faculty | 4 | (Abdulmalik et al., 2014; Foster et al., 2018; Goldstone & Ntuli, 2016; Matovu et al., 2011) |
| Ensure country ownership | 2 | (Foster et al., 2018; Goldstone & Ntuli, 2016) |
| Train participants to become future mentors and faculty | 2 | (Goldstone & Ntuli, 2016; Muhimpundu et al., 2018) |
| Anticipate resource constraints in the setting | 1 | (Kebede et al., 2010) |
