# Supplementary figures and images for: Interventions to strengthen the leadership capabilities of health professionals in Sub-Saharan Africa: a scoping review
Source: Health Policy Plan. 2020 Dec 13;36(1):117–33. doi: 10.1093/heapol/czaa078 (PMC7938510; doi:10.1093/heapol/czaa078)

**Table 3: Teaching and learning methods of the leadership development programmes**


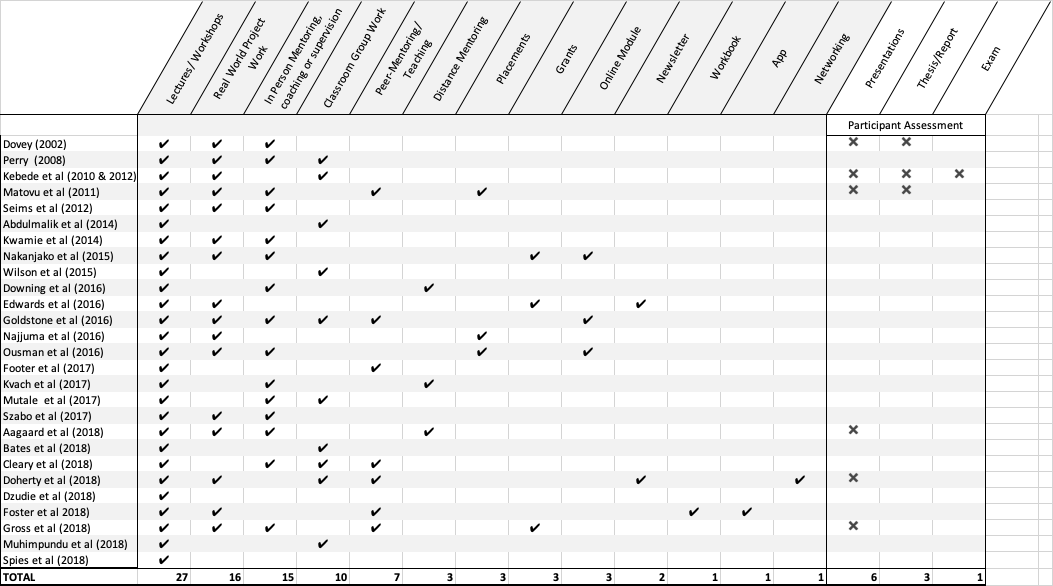

Supplement: czaa078_Supplementary_Data [file czaa078_supplementary_data.zip › Table 3.docx]
